# Patient Posture Monitoring System Based on Flexible Sensors

**DOI:** 10.3390/s17030584

**Published:** 2017-03-13

**Authors:** Youngsu Cha, Kihyuk Nam, Doik Kim

**Affiliations:** 1 Center for Robotics Research, Korea Institute of Science and Technology, Seoul 02792, Korea; todream623@naver.com (K.N.); doikkim@kist.re.kr (D.K.); 2 Department of Mechanical Engineering, Hanyang University, Seoul 04763, Korea

**Keywords:** flexible sensor, patient cloth, piezoelectric material, real-time monitoring

## Abstract

Monitoring patients using vision cameras can cause privacy intrusion problems. In this paper, we propose a patient position monitoring system based on a patient cloth with unobtrusive sensors. We use flexible sensors based on polyvinylidene fluoride, which is a flexible piezoelectric material. The flexible sensors are inserted into parts close to the knee and hip of the loose patient cloth. We measure electrical signals from the sensors caused by the piezoelectric effect when the knee and hip in the cloth are bent. The measured sensor outputs are transferred to a computer via Bluetooth. We use a custom-made program to detect the position of the patient through a rule-based algorithm and the sensor outputs. The detectable postures are based on six human motions in and around a bed. The proposed system can detect the patient positions with a success rate over 88 percent for three patients.

## 1. Introduction

Recently, monitoring technologies geared to the care of patients and the elderly have received considerable attention [1,2,3]. Monitoring systems can be adapted for management applications in hospitals, nursing facilities, and the elderly that live alone [3,4,5]. These systems can be managed by only a few nurses and have great benefits, such as reducing healthcare cost and providing efficient service [6,7].

In this paper, we propose a patient posture monitoring system based on flexible sensors. The easiest way to monitor people is to use vision cameras [8]. It can be used for fall detection [9,10], sleep monitoring [11,12], breathing detection [13,14], depression detection [15,16], measuring vital signs [17,18], and posture detection [19,20]. However, they can have blind spots because of the camera’s position. In addition, vision-based monitoring systems have a privacy problem [8,21].

An alternative method is using sensing devices such as accelerometers and gyroscopes [22]. Without the invasion of user privacy, these devices can monitor human walking [23,24,25], posture transition [26,27,28], and daily activity [29,30,31]. Although the sensors protect privacy, detailed posture detection can be difficult. To collect sufficient data on the patient’s position and posture, many sensors would be attached to the user. Further, the sensors have mostly rigid cases, which result in inconvenience to the users.

The feasibility of new monitoring systems using flexible sensors are considered as an option [32,33]. The flexible sensors can be developed by microfabrication process [34,35,36,37,38], textile weaving [39,40], or commercial materials [41]. The sensors are adapted for snoring detection system [41], mobility pattern research of a person on a bed [42], human motion sensing [43,44], and real-time health and fitness monitoring [45].

For our system, we use flexible piezoelectric sensors consisting of polyvinylidene fluoride (PVDF) [46,47,48], which is the one of the most flexible piezoelectric materials [49,50,51]. The piezoelectric sensors are installed into the parts close to the knee and hip of the loose patient cloth to be worn. When the knee and hip are bent, the sensors generate voltage signals that are measured by the analog–digital converter in a micro-controller module. The measured signals are wirelessly sent to a computer with a posture monitoring program. Therein, by the use of a rule-based algorithm and the processed sensor outputs, we detect six human motions occurring in and around the bed of three patients who are wearing the cloth.

From a practical point of view, this work addresses the untapped research question of sensing human motions from sensors embedded in a loose cloth. From a methodological point of view, the main contributions of this effort are: (i) developing a patient posture monitoring system using the loose patient cloth and flexible piezoelectric sensors; (ii) performing a thorough experimental campaign to assess the performance of the monitoring system; and (iii) conducting an analysis of the sensing characteristics of the flexible sensors in the given human motion conditions.

This paper is organized as follows. In Section 2, we introduce the proposed system setup with the description about the patient cloth, flexible sensor, and sensing module. In Section 3, we show and discuss the sensor outputs during the six transition motions between four positions. The decision method using the sensor outputs for the posture detection is reported in Section 4. In Section 5, the patient posture monitoring system is experimentally demonstrated. The conclusions are summarized in the final section.

## 2. System Setup

**Figure 1:**
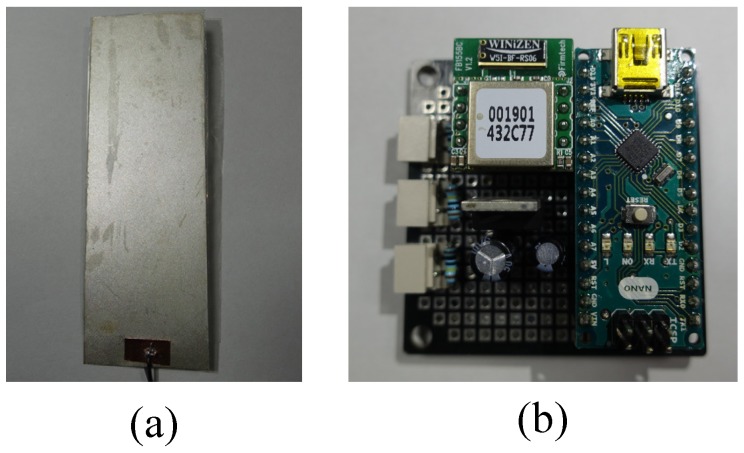
Pictures of the (**a**) flexible sensor and (**b**) sensing module.

We fabricate flexible sensors consisting of PVDF (produced by Measurement Specialties) and Mylar [47,48]. A PVDF layer with the size of $75\times 25\times 0.11\;{\mathrm{mm}}^{3}$ was attached to a Mylar sheet with $70\times 25\times 0.1\;{\mathrm{mm}}^{3}$ using 3M DP460 epoxy. The capacitance of the PVDF layer was 2.66nF, measured using a FLUKE-17B+ digital multimeter. To sense electrical signals from the PVDF, conductive adhesive 3M copper foil tape 1181 was utilized with copper electrodes on both sides. Two wires were soldered to access the copper electrodes, and were connected to a sensing module. Each component in the sensing module was soldered on a universal printed circuit board (PCB) with the size of $50\times 50\;{\mathrm{mm}}^{2}$. Figure 1 displays the fabricated flexible sensor and sensing module.

**Figure 2:**
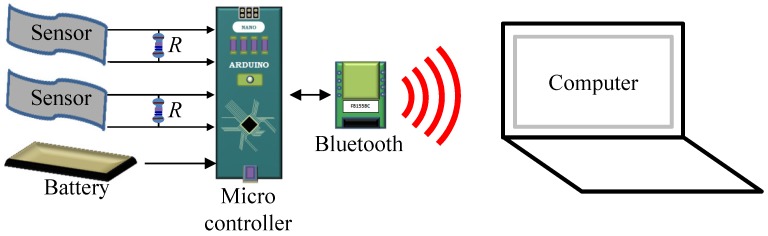
Schematic of the sensing system consisting of two flexible sensors and one sensing module.

The sensing module was utilized to record the voltages from the sensors and to transfer the data wirelessly. The voltage signals were measured through 10-bit analog–digital converters of an Arduino Nano micro-controller, with load resistors, R=1MΩ (see Figure 2). One end of the load resistor was connected to a 2.5V source generated by a SPX1587AU-2.5 regulator. This connection makes the sensor outputs maintain a positive value, which can be detected in the analog–digital converters. Ideally, the sensor output at 2.5V is 512 (binary number 1000000000). The digitalized sensor outputs and measured time are transferred to a computer through a FB155BC Bluetooth unit. The sensor outputs are recoded and sent at 90∼100Hz. The sensing module is supported by a lithium polymer battery with the voltage output of 7.4V and a capacity of 650mAh.

**Figure 3:**
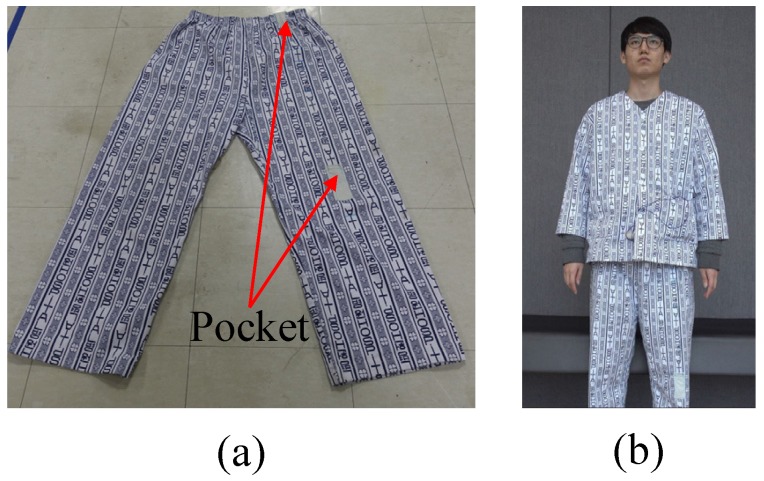
Pictures of the (**a**) patient pants with two pockets for the flexible sensors and (**b**) patient wearing the sensing system.

The sensing system was installed in a patient cloth. In particular, two flexible sensors were attached to two parts of the patient pants using pockets, as shown in Figure 3a. The pockets were positioned in the left knee and hip parts. Figure 3b displays a male student (Patient1) wearing the patient cloth. The sensing module was put into a vest pocket of the patient’s upper garment.

## 3. Patient Posture and Sensor Output

**Figure 4:**
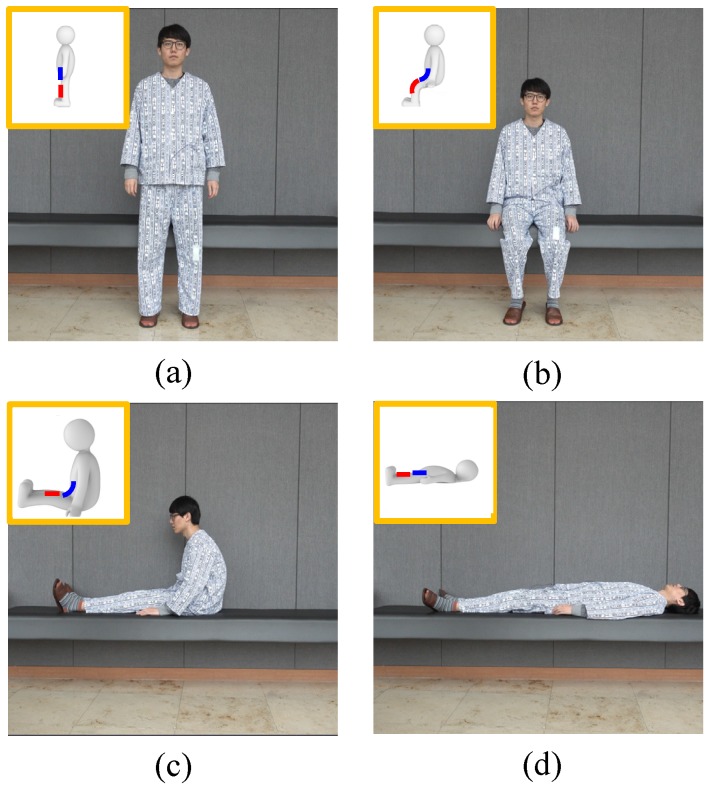
Patient postures with the (**a**) standing; (**b**) sitting; (**c**) sitting knee extension; and (**d**) supine positions. In the inset, the red and blue lines are the sensor shapes at the knee and hip, respectively.

We focus on the four positions of a patient in and around a bed. In particular, they are standing, sitting, sitting knee extension, and supine poses, as shown in Figure 4. The standing pose is when a patient is in a vertical position. The sitting position means that a patient sits with bent knees on a bed. The sitting knee extension posture is to sit with the knees outstretched on a bed. The supine pose is to lie horizontally on a bed. In addition, we show the shapes of the sensors as each pose in the inset of Figure 4.

**Figure 5:**
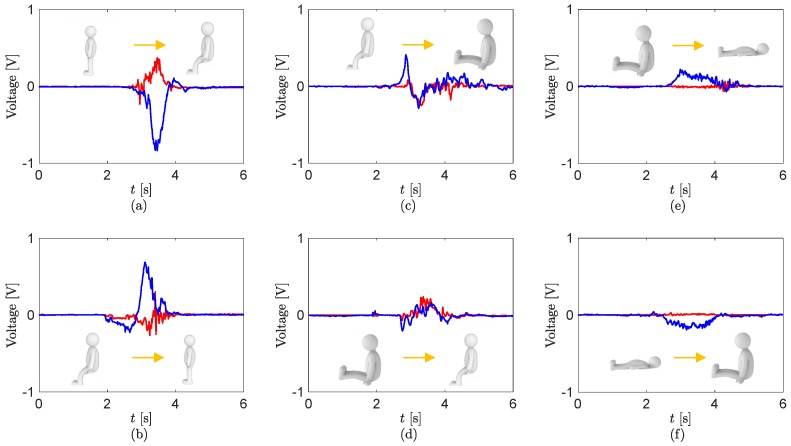
Digitalized sensor outputs at the transition (**a**) from the standing to sitting position; (**b**) from the sitting to standing position; (**c**) from the sitting to sitting knee extension position; (**d**) from the sitting knee extension to sitting position; (**e**) from the sitting knee extension to supine position; and (**f**) from the supine to sitting knee extension position. The red and blue lines are the data of the sensor at the knee and hip, respectively.

Figure 5 displays the time traces of the sensor output voltages at the knee and hip during the six transitions between the four positions. Specifically, when the transition from the standing to sitting position occurs, the sensors at the knee and hip are bent along with the joints. Interestingly, the phase of the sensor outputs are opposite because of the difference of each bending direction (see Figure 4a). Further, the hip value is higher than the knee; that is, the bending at the hip is larger. When the pose of the patient comes back to the standing position, the sensor outputs in Figure 5b are the counter images of Figure 5a. Figure 5c displays the signals when the knee is extended while sitting. This signal at the knee has mostly negative values, whereas the one at the hip oscillates between positive and negative values. In Figure 5d, we observe the reverse phase of that case from the sitting knee extension to the bent knee position. Figure 5e,f display the positive and negative values from only the hip sensor by the extension and bending of the joint. In addition, we observe that the pose transitions happen for approximately 2 s.

## 4. Decision Method

To detect the patient’s postures from the sensor outputs in Figure 5, we do data processing by using C# language. The main processing steps are described as follows.

**Figure 6:**
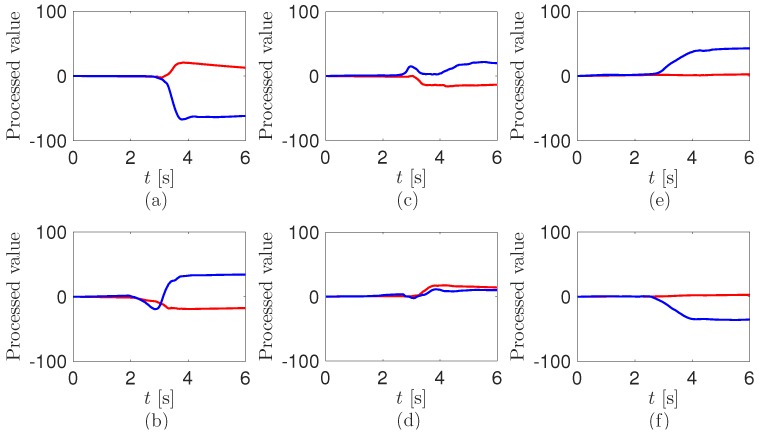
Signal processed values from the sensor outputs at the transition (**a**) from the standing to sitting position; (**b**) from the sitting to standing position; (**c**) from the sitting to sitting knee extension position; (**d**) from the sitting knee extension to sitting position; (**e**) from the sitting knee extension to supine position; and (**f**) from the supine to sitting knee extension position. The red and blue lines are the data of the sensor at the knee and hip, respectively.

**Figure 7:**
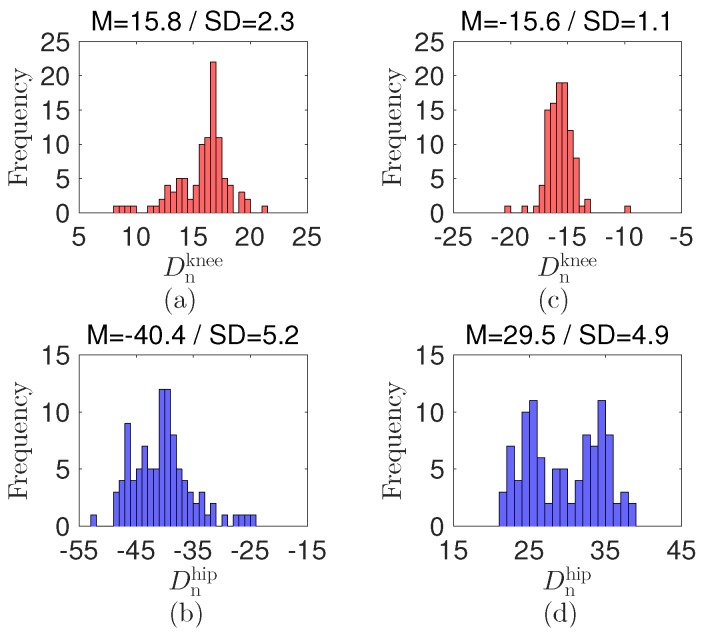
Histograms of the difference $D_{n}$. (**a**) $D_{n}^{\mathrm{knee}}$ and (**b**) $D_{n}^{\mathrm{hip}}$ at the transition from the standing to sitting. (**c**) $D_{n}^{\mathrm{knee}}$ and (**d**) $D_{n}^{\mathrm{hip}}$ at the transition from the sitting to standing. “M” and “SD” indicate their mean and standard deviation.

**Figure 8:**
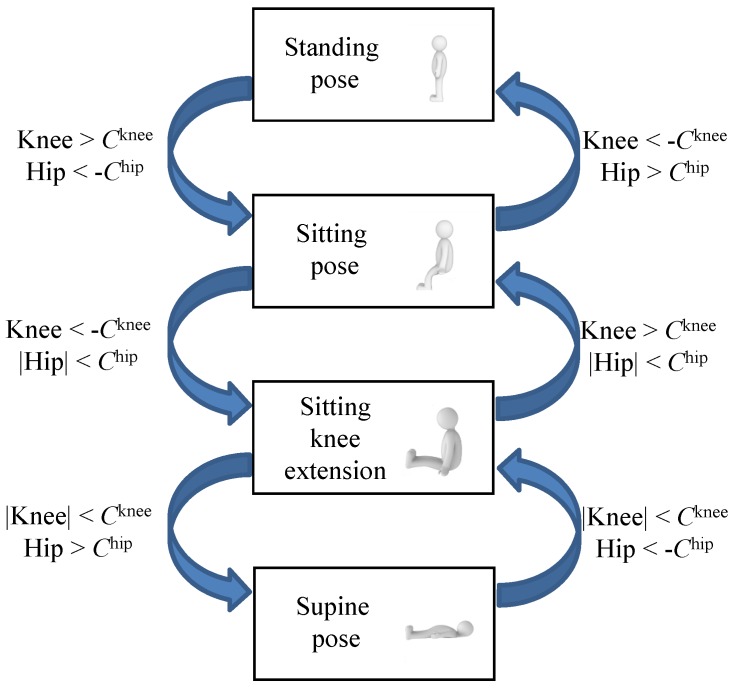
Flow chart for posture decisions.

**Step 1:**  The offsets of the digitalized sensor outputs in Figure 5 have removed. We use the average value of 9000 in the dataset as the offset. It is mathematically described as
  (1) $$y_{n}=x_{n}-\frac{\sum_{m=n-8999}^{n}x_{m}}{9000}$$
  where $x_{n}$ is the digitalized sensor output, and $y_{n}$ is the value without the offset.
**Step 2:**  The values after removing the offset are integrated as the time
  (2) $$z_{n}=z_{n-1}+y_{n}\times \Delta t_{n}$$
  where $z_{n}$ is the integrated value and $\Delta t_{n}$ is the time interval.
  The processed values from the sensor outputs in Figure 5 are shown in Figure 6. In Figure 6a, the positive knee value and the negative hip value are saturated. Further, they have the opposite values in Figure 6b. This is due to the characteristics of the piezoelectric sensor outputs, of which raw voltage and integration at a load resistance may be proportional to the angular velocity and angle, respectively [52]. The profiles of the processed values from the knee in Figure 6c,d are the same as in Figure 6a,b, respectively. The values at the hip in Figure 6c,d are oscillated and converged to much smaller ones than in Figure 6a,b. In Figure 6e,f, while the values at the knee are not moving, the ones at the hip are saturated to the larger positive and negative values, respectively. The converged values at the hip are slightly more decreased than in Figure 6a,b. Reasons for the decreases may be ascribed to the changes of the bending of the pants during the turning motion between the sitting position and sitting knee extension position.
**Step 3:**  We use a rule-based algorithm [53,54] to decide the postures of the patient wearing the cloth with the sensors. Specifically, the bending and extension of the knee and hip at $90^{\circ }$ of the sensors in the patient’s pants can be detected from the processed values in Figure 6. We compare the difference between the current processed value and the previous one before 200 counts; that is,
  (3) $$D_{n}=z_{n}-z_{n-199}$$
  (4) $$\begin{array}{c} \left\{\begin{array}{cc} D_{n}^{\mathrm{knee}}>C^{\mathrm{knee}} & \mathrm{knee}\;\mathrm{bending}\\ |D_{n}^{\mathrm{knee}}|<C^{\mathrm{knee}} & -\\ D_{n}^{\mathrm{knee}}<-C^{\mathrm{knee}} & \mathrm{knee}\;\mathrm{extension} \end{array}\right. \end{array}$$
  (5) $$\begin{array}{c} \left\{\begin{array}{cc} D_{n}^{\mathrm{hip}}>C^{\mathrm{hip}} & \mathrm{hip}\;\mathrm{extension}\\ |D_{n}^{\mathrm{hip}}|<C^{\mathrm{hip}} & -\\ D_{n}^{\mathrm{hip}}<-C^{\mathrm{hip}} & \mathrm{hip}\;\mathrm{bending} \end{array}\right. \end{array}$$
  where $D_{n}^{\mathrm{knee}}$ and $D_{n}^{\mathrm{hip}}$ are the differences at the knee and hip, respectively. $C^{\mathrm{knee}}$ and $C^{\mathrm{hip}}$ are the decision values at the knee and hip, respectively.
  The decision values for the detection are selected as $C^{\mathrm{knee}}=7.5$ and $C^{\mathrm{hip}}=20$ by preliminary tests with each having 100 repetitions between the standing and the sitting positions; see the histogram and statistical data in Figure 7. The decision values are $C^{\mathrm{knee}}$/$-C^{\mathrm{knee}}$ and $-C^{\mathrm{hip}}$/$C^{\mathrm{hip}}$ at the knee and hip during the joint bending/extension, respectively. Notably, the decision values and signs at the knee and hip are different. The value gap can be attributed to the effect of each joint structure, which results in different mechanical displacement of the sensor. The variations under the decision values expect that the bending and extension of the joints are smaller than $90^{\circ }$. The opposite sign results from the different bending direction at the knee and hip. The decision values may differ as the body structure of the patient varies.
**Step 4:**  By using the previous posture state and the decisions in Step 3, we predict the current position of the patient; that is,
  (6) $$S_{n}=f\left(D_{n}^{\mathrm{knee}},D_{n}^{\mathrm{hip}},S_{n-1}\right)$$
  where $S_{n}$ is the posture state and f(·) is the decision function.
  Figure 8 displays the flow chart to decide the position of the patient. For example, when $D_{n}^{\mathrm{knee}}$ is under $-C^{\mathrm{knee}}$ and $D_{n}^{\mathrm{hip}}$ is higher than $C^{\mathrm{hip}}$ at the sitting pose, the transferred pose is standing. In the program, we use the standing position as the initial state.

## 5. System Operation

**Figure 9:**
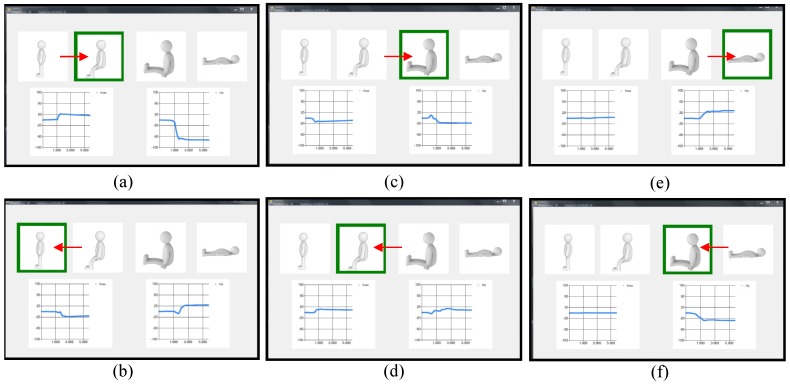
Snapshots of the computer screen showing the estimated patient postures and signal processed values from the sensor outputs at the transition (**a**) from the standing to sitting position (i); (**b**) from the sitting to standing position (ii); (**c**) from the sitting to sitting knee extension position (iii); (**d**) from the sitting knee extension to sitting position (iv); (**e**) from the sitting knee extension to supine position (v); and (**f**) from the supine to sitting knee extension position (vi). The arrows are added to represent the transitions from the previous positions.

In this section, we demonstrate the operation of the patient posture monitoring system. The student wearing the patient cloth with the sensors goes through the six motions (i) from the standing to sitting position; (ii) from the sitting to standing position; (iii) from the sitting to sitting knee extension position; (iv) from the sitting knee extension to sitting position; (v) from the sitting knee extension to supine position; and (vi) from the supine to sitting knee extension position. Figure 9 displays the computer screen of the patient posture monitoring system for the six transitions. The green borders show the current postures of the patient. The left and right bottom graphs in Figure 9 display the processed values at the knee and hip, respectively. The operation of the patient posture monitoring system is displayed in Video S1 as a supplementary material.

To assess the patient posture monitoring system, a total of 900 experiments from three students wearing the patient clothes with different size (50 per each motion and person) were performed. We use the decision values for Patient2, $C^{\mathrm{knee}}=10$ and $C^{\mathrm{hip}}=20$, and Patient3, $C^{\mathrm{knee}}=7.5$ and $C^{\mathrm{hip}}=10$, selected by preliminary tests. Table 1 shows the success rate of the six transition motions of the three patients. All motions of three patients were detected with a success rate over 88%. A few times, we failed to detect the motions because of low sensor values. In motion cases (i) and (ii), the position of the knee sensor during bending or releasing was sometimes misaligned. In motion cases (iii) and (iv), this may be ascribed to the release of the hip sensor for turning motion between the sitting and sitting knee extension positions. In motion cases (v) and (vi), the hip sensor could have a low value because of the subsequent release of the bent hip sensor and misalignment.

## 6. Conclusions

In this paper, we demonstrated a patient posture monitoring system based on a patient cloth with embedded flexible piezoelectric sensors. The patient cloth was fitted loosely, and the flexible sensors were positioned in the knee and hip parts of the cloth. The sensor generated electrical responses corresponding to the bending and extension of each joint. The sensor outputs were wirelessly transferred to a computer with a custom-made program. The data accurately predicted the position of the patient through a rule-based algorithm after processing the electrical signals. We tested the monitoring system with six motions between four positions that can happen in or around a bed. Our system operation was working well with a success rate over 88% for three patients. The demonstration of the system suggests a patient monitoring system using unobtrusive wearable sensors. Without a vision camera, this monitoring system can monitor the position of patients while protecting their privacy. This has beneficial uses in hospitals and nursing facilities. Future studies will focus on the performance evaluation of the system through adaptation in various test subject groups and comparison with other devices. 

## Figures and Tables

**Table 1:** Success rate of the patient posture monitoring system.

Motion	(i)	(ii)	(iii)	(iv)	(v)	(vi)
Patient1 (M–23 years–185 cm–72 kg) (%)	100	100	92	96	90	100
Patient2 (M–24 years–171 cm–67 kg) (%)	88	96	96	98	94	98
Patient3 (F–21 years–160 cm–51 kg) (%)	98	96	88	94	100	100

## Supplementary Materials

The following are available online at http://www.mdpi.com/1424-8220/17/3/584/s1, Video S1: Operation of the patient posture monitoring system.
